# Family caregiving challenges in primary palliative care for older people with multimorbidity in Malawi – A cross-sectional qualitative study

**DOI:** 10.4102/phcfm.v18i1.5490

**Published:** 2026-09-30

**Authors:** Duncan P. Kwaitana, Dorothee van Breevoort, Modai C. Mnenula, Kennedy Nkhoma, Richard Harding, Maya J. Bates

**Affiliations:** 1 Department of Family Medicine, School of Medicine and Oral Health, Kamuzu University of Health Sciences, Blantyre, Malawi; 2 Cicely Saunders Institute of Palliative Care Policy and Rehabilitation, Faculty of Nursing, Midwifery and Palliative Care, King’s College London, London, United Kingdom

**Keywords:** multimorbidity, older people, primary care, primary palliative care, caregiver, Malawi

## Abstract

**Background:**

Global population ageing has led to a rise in older people living with multimorbidity. In low-resource settings, they rely heavily on family members for primary palliative care (PPC) and daily support, placing substantial demands on caregivers.

**Aim:**

To explore the challenges experienced by family members providing PPC to older people living with multimorbidity in Malawi.

**Setting:**

Peri-urban district of Malawi at two primary health care facilities and within participants’ homes.

**Methods:**

A cross-sectional qualitative design was employed between January 2021 and March 2021. Fifteen caregivers were purposively selected. In-depth interviews were conducted either at the health facility or in participants’ homes, according to their preference. Data were analysed inductively to generate themes, supported by NVivo 12 Pro software.

**Results:**

Four overarching themes and nine sub-themes emerged: (1) Complex decision-making dynamics (family hierarchy and interdependence); (2) Demanding caregiving responsibilities (patient-related care and household duties); (3) Consequences for caregiver well-being (psychosocial and spiritual impacts); (4) Expressed needs to support caregiving roles (person-centred care, strengthened community-based primary health care services, and consistent availability of essential medicines).

**Conclusion:**

Family caregivers are an integral component of PPC in providing PPC for older people living with multimorbidity. Interventions should address the practical and emotional challenges they encounter in their caregiving roles.

**Contribution:**

This study provides context-specific evidence on the multidimensional challenges faced by family caregivers of older people with multimorbidity in Malawi and underscores the need to strengthen community-based primary care and integrate caregiver support into palliative care services in similar low-resource settings.

## Introduction

The global population is ageing, with an estimated 901 million older people – representing 12.3% of the world’s population.^1^ By 2050, projections suggest that 80% of older people will live in low- and middle-income countries (LMICs).^2^ Although the definition of older age remains contested, many high-income countries conventionally define it primarily on the basis of chronological age, typically as 60 years or 65 years and above.^3^ However, for policy development and cross-cultural comparability in African research contexts, an age threshold of 50 years is commonly used to define older age.^4^ In sub-Saharan Africa (SSA), this demographic shift is accompanied by a growing prevalence of multimorbidity, defined as the co-existence of two or more chronic conditions in one individual.^5,6^ While multimorbidity has long been recognised in primary care populations in high-income countries,^7^ it is now increasingly prevalent worldwide as populations age.^8^ The World Health Organization (WHO), through the Astana Declaration, highlights the central role of primary care in delivering integrated services and in creating ‘enabling and health-conducive’ environments for individuals and communities.^9^ Multimorbidity presents significant challenges for affected individuals, their families and already strained health systems.^10^ Yet in many LMICs, vertical (single-disease-focused) health programmes limit the delivery of integrated care for people with multimorbidity.^10,11^

As populations age and non-communicable diseases (NCDs) increase, primary palliative care (PPC) is becoming more relevant.^12,13^ Primary palliative care has been defined as the integrated and holistic management of disease and symptoms by primary health care workers for people in their local communities.^14^ It generally refers to the provision of basic palliative care by non-specialist healthcare professionals.^15^ In addition to healthcare providers, family members play a critical role in supporting individuals experiencing serious health-related suffering.^16,17,18,19^ Their contribution as informal, non-specialist caregivers is particularly important for older people, who often depend on family members for daily care and support. In resource-limited settings, where specialist palliative care services and healthcare workforce capacity may be limited, family caregivers often help bridge gaps in care provision.^20^ Caregivers are typically informal providers of physical, social, practical and emotional support.^21^

In LMICs, however, family caregivers face numerous socioeconomic challenges that make their role difficult and overwhelming.^22,23^ The impact is often long-term, resulting in caregiver morbidity, reduced work capacity and diminished quality of life.^6^ Despite the increasing prevalence of multimorbidity,^10,22,24^ limited evidence exists on the challenges faced by caregivers of older people in SSA as they provide PPC. Generating such evidence is critical for informing and strengthening holistic models of PPC delivery. Without addressing these challenges, the rising burden of multimorbidity in older populations across SSA and other LMICs may undermine progress towards universal health coverage (UHC). The WHO defines UHC as ensuring that:

[*A*]ll people and communities can use the promotive, preventive, curative, rehabilitative and palliative health services they need, of sufficient quality to be effective, while also ensuring that the use of these services does not expose the user to financial hardship.^25^

While previous studies have documented the general burden experienced by family caregivers of people living with serious illnesses in LMICs, most have focused on single conditions such as advanced cancer or human immunodeficiency virus (HIV) or have examined caregiving within high-income settings.^17,19,22,23^ Evidence on the experiences of family caregivers supporting older people living with multimorbidity within PPC in SSA remains scarce.^17,19^ This study addresses this important knowledge gap by providing one of the first qualitative investigations of family caregiving among older people living with multimorbidity receiving PPC in Malawi. The findings generate context-specific evidence to inform the development of integrated, person-centred PPC models, caregiver support interventions and health policies for ageing populations in SSA.^26,27^

## Research methods and design

### Study design

This was a cross-sectional qualitative study that adopted the interpretivist approach^28^ to explore the challenges of family caregivers of older people (50 years or older) in relation to their primary palliative caregiving role. Data were collected through in-depth, face-to-face interviews. The study is reported in accordance with the Consolidated criteria for Reporting Qualitative Research (COREQ) checklist (Online Appendix 1).^29^ The data used in this study originate from a previously described study and were reanalysed for the present investigation.^26^

### Study setting

This study was nested within a multi-country mixed-methods project conducted in Malawi, Ghana and Zimbabwe to develop a model of PCC for older people with serious multimorbidity.^26,27,30^ The present paper reports findings from a two-site study in Malawi, conducted in Mangochi District, one of the largest districts in the country’s southern region. Mangochi, classified as peri-urban, had an estimated population of 1 346 740 in 2023.^31^ Mangochi District Hospital is a 500-bed public secondary-level facility and the only referral hospital serving the district. Although it functions as a referral hospital, it also provides primary health care services, which are largely delivered by nurses and clinical officers. In addition to outpatient and inpatient palliative care services, the hospital supports PPC through community nurses working alongside community volunteers and family caregivers to provide home-based care (HBC).

Lungwena Health Centre, one of the district’s 42 health centres, was also designated as the second Malawi study site within the larger multi-country study. At the time of the study, the facility was led by a medical assistant and primarily provided general outpatient services, with no dedicated palliative care or NCD clinics. Patients within its catchment area accessed PPC through the HBC team based at Mangochi District Hospital, supported by community volunteers and family caregivers.

### Recruitment procedure

Caregivers were purposively selected using medical records of older people diagnosed with multimorbidity from the following outpatient clinics at Mangochi District Hospital: (1) palliative care, (2) NCDs, and (3) physiotherapy. At Lungwena Health Centre, caregivers were recruited from the general outpatient clinic if the medical record indicated that the older person under their care had a diagnosis of multimorbidity and was receiving PPC services.

Caregivers who accompanied older people to the hospital were invited to participate, recognising that, given their caregiving role, they could provide deeper insights into the lived experience of multimorbidity. All participants provided PPC at home. Participants were informed about the study objectives, procedures and their right to withdraw from the study at any time without any negative consequences. Those who expressed interest and met the inclusion criteria were given information sheets in local languages. For participants who were unable to read or write, two research assistants (one male and one female), both with a high school education, prior training and experience in qualitative research, and fluent in both Chichewa and Chiyao, read the information sheet aloud in the presence of an impartial witness.

### Inclusion criteria

Caregivers were defined as unpaid, informal providers of one or more physical, social, practical and emotional caregiving tasks within PPC.^14^ In terms of their relationship to the patient, they may be a friend, partner, ex-partner, sibling, parent, or other blood or non-blood relative.^32^ Family caregivers of older people living with multimorbidity were recruited if they were at least 18 years of age and provided care for the older person (aged at least 50 years of age) with a diagnosis of two or more serious conditions such as HIV, stroke, diabetes, hypertension and chronic obstructive pulmonary disease (COPD).

### Data collection

Between January 2021 and March 2021, qualitative semi-structured interviews were conducted with 15 family caregivers. Data were collected through semi-structured interviews guided by a predetermined topic guide (Online Appendix 1). The guide was developed based on palliative care models and evidence on PPC in LMICs. It explored: (1) patient and family needs (physical, psychological, social and spiritual, consistent with the WHO definition of palliative care);^18^ (2) current primary care practices for older people with multimorbidity; (3) potential barriers and facilitators; and (4) system changes and training needs required to deliver PPC.

The interview guide was developed by the research team, which included an experienced Malawian qualitative researcher (Kennedy Nkhoma, PhD, male), a Malawian palliative care clinician (Duncan P. Kwaitana, MSc, male), a Malawian Family Medicine physician (Modai C. Mnenula, FAMMED, male), a United Kingdom (UK) palliative care research academic and social worker (Richard Harding, PhD, male), and a Dutch researcher with expertise in public health (Dorothee van Breevoort, PhD, female). It was further refined after two pilot interviews. Data collection continued until the team collaboratively decided that sufficient information power reached, with no new themes emerged in three consecutive interviews and existing themes had been sufficiently and thoroughly explored.^33^

Interviews were audio-recorded and held either in a private room within the health facility or at participants’ homes, depending on their preference. All caregivers who were purposively sampled consented to participate.

### Data analysis

Interviews were transcribed verbatim in the local language and subsequently translated into English by the research assistants. The quality and accuracy of translations were reviewed by Duncan P. Kwaitana (fluent in English, Chiyao and Chichewa), who re-read portions of all transcripts.

The English transcripts were uploaded into NVivo 12 Pro (Lumivero, Denver, CO, United States [US]) for analysis. After familiarisation with the data, the research team, comprising three Malawian researchers (Kennedy Nkhoma, Duncan P. Kwaitana, Modai C. Mnenula) and one Dutch researcher (Dorothee van Breevoort), independently conducted an inductive thematic coding. The team then met to consolidate their analyses into a unified thematic framework. The final analysis focused primarily on understanding the role of family caregivers and the challenges they experienced while providing PPC to older people living with multimorbidity. The resulting codes were subsequently organised and mapped to the identified themes using NVivo software.

### Ethical considerations

Ethical clearance to conduct this study was obtained from the University of Malawi College of Medical Research and Ethics Committee (No. P.08/20/3108). Ethical approval was obtained from King’s College Research Ethics Committee (Ref: HR-19/20-18524) and the College of Medicine Research and Ethics Committee (COMREC) (Ref: P.08/20/3108). Permission to collect data from the participants was obtained from the District Health Office. Participants received both verbal and written information about the study. The consent form was read aloud, and informed consent was obtained by signature or thumbprint. Data were collected anonymously and encrypted with password, ensuring that only authorised members of the research team could access them. This approach safeguards the confidentiality of all information shared by participants.

## Results

### Participant characteristics

Table 1 presents the demographic characteristics of the older people for whom family caregivers provided PPC. The older patients ranged in age from 50 years to 75 years, with a median age (interquartile range [IQR]) of 62 (53–67) years. Of the 15 older patients, eight were men and seven women. The older patients were diagnosed with hypertension and diabetes (*n* = 5), stroke and hypertension (*n* = 3), HIV and cancer (*n* = 2), hypertension and cancer (*n* = 2), stroke, hypertension and diabetes (*n* = 2), and stroke and diabetes (*n* = 1).

**TABLE 1:** Demographic characteristics of older people with multimorbidity receiving primary palliative care.

Variable	Patients		Median	IQR
	Frequency (*n*)	%		
**Age range (years)**	50–75	-	62	53–67
**Gender**				
Male	8	53	-	-
Female	7	47	-	-
**Education**				
No education	6	40	-	-
Primary school	2	13	-	-
Secondary school	6	40	-	-
Tertiary or college	1	7	-	-
**Religion**				
Islam	10	67	-	-
Christianity	5	33	-	-
**Occupation**				
Employed	1	7	-	-
Not employed	7	47	-	-
Farmer	6	40	-	-
Business	1	7	-	-
**Diagnosis**				
Hypertension and diabetes	5	33	-	-
Stroke and hypertension	3	20	-	-
HIV and cancer	2	13	-	-
Hypertension and cancer	2	13	-	-
Stroke, hypertension and diabetes	2	13	-	-
Stroke and diabetes	1	7	-	-

IQR: interquartile range; HIV, human immunodeficiency virus.

We recruited 15 caregivers (see Table 2). Interviews lasted between 50 min and 90 min. Participants’ ages ranged from 21 years to 66 years, with the majority being female (87%). Nearly half of the caregivers (47%) were farmers by occupation. Five categories of relationships between patients and caregivers were identified: sibling, parent, child, wife and husband. The category of ‘child’ included biological children, as well as nephews and nieces, as in many African contexts extended family structures and caregiving norms often recognise these relatives as child caregivers.^34^ Consequently, participants C009, C010 and C014 were classified as child caregivers for analytical purposes. On average, participants had provided care for the older family member for 6 years, with durations ranging from 2 months to 15 years.

**TABLE 2:** Characteristics of family caregivers providing primary palliative care (*N* = 15).

Variable	*n*	%	Median	IQR
**Age range (years)**	21–66	-	45	29–53
**Gender**				
Male	2	13	-	-
Female	13	87	-	-
**Education**				
No education	2	13	-	-
Primary school	9	60	-	-
Secondary school	2	13	-	-
Tertiary or college	2	13	-	-
**Religion**				
Islam	9	60	-	-
Christianity	6	40	-	-
**Occupation**				
Employed	0	-	-	-
Not employed	3	20	-	-
Casual temporary or housewife	3	20	-	-
Farmer	7	47	-	-
Business	1	7	-	-
Student	1	7	-	-
**Relationship with patient**				
Sibling	3	20	-	-
Wife	4	27	-	-
Parent	3	20	-	-
Husband	1	7	-	-
Child	4	27	-	-

IQR: interquartile range.

### Challenges experienced by family caregivers in providing primary palliative care

Four themes and nine sub-themes emerged, capturing the challenges experienced by caregivers of older people living with multimorbidity while providing primary PPC. These themes and sub-themes are summarised in Table 3.

**TABLE 3:** Summary of themes and sub-themes derived from analysis of the data.

Major themes	Sub-themes
1 Complex decision-making dynamics	1.1 Family hierarchy 1.2 Interdependence
2 Demanding caregiving responsibilities	2.1 Responsibilities for patient-related care 2.2 Responsibilities for the household
3 Consequences on caregiver well-being	3.1 Spiritual 3.2 Psychosocial
4 Expressed needs for fulfilling caregiver roles	4.1 Need for person-centred care 4.2 Strengthened community-based primary care services 4.3 Availability of essential medicines

#### Theme 1: Complex decision-making dynamics

**Sub-theme 1.1: Family hierarchy:** Family caregivers reported experiencing challenges within the household when the head of the family became ill. Previously, the head of the family played a central role in decision-making and providing for family needs, but this dynamic shifted once they became the recipient of PCC:

‘Things get complicated sometimes because he is the one who is supposed to sort things out but due to his illness there is lack of understanding. This is because the elder person of the family is the one who is sick. He brings togetherness and sort things out.’ [*indicating that the illness rendered the head of the family incapable of controlling household conflicts*] (C014, family caregiver, male, 21 years)

**Sub-theme 1.2: Interdependence:** In addition, some family caregivers reported assuming the role of decision-maker regarding PPC for their older relative. In consultation with other family members, they determined when it was necessary for the patient to seek hospital care:

‘It’s me who takes a big part, because most of the time the patient does not think properly, he just knows that he is sick, any pain he feels he says is because of the same sickness then this is up to the guardian to think what to do, so that the problem should not become worse.’ (C005, family caregiver, female, 53 years)

#### Theme 2: Demanding caregiving responsibilities

**Sub-theme 2.1: Responsibilities for patient-related care:** Caregivers reported providing daily PPC at home with little or no assistance, undertaking responsibilities such as bathing, cleaning the house and surrounding compound, preparing meals, and assisting patients with mobility and toileting. They described these tasks as overwhelming for one person, with some explaining that they were unable to attend to other household responsibilities because the patient required continuous supervision and could not be left unattended:

‘I do take care of him, escort him to the hospital, wash clothes for him, give him a wash so he looks good though he is sick, fetch food for him to eat, that’s the work. Things have been greatly affected since my husband became ill.’ (C002, family caregiver, female, 45 years)

Additionally, family caregivers reported being responsible for keeping track of the patient’s clinic appointments and often accompanied them to the hospital to provide support during visits:

‘When we visit the hospital, I make sure that my mother gets help. For instance, if the doctors are not available, I go look for them in order for my mother to be helped.’ (C009, family caregiver, female, 50 years)

**Sub-theme 2.2: Responsibilities for the household:** Caregivers expressed concerns about food security, explaining that they often lacked sufficient time to cultivate their fields because most of their time was devoted to providing PPC, with limited opportunities to transfer caregiving responsibilities to another person. As a result, households experienced reduced income, as caregivers were unable to engage in income-generating activities. These financial constraints also affected children’s education, either because families could no longer afford school-related expenses or because children had to take on caregiving responsibilities:

‘This [*caregiving*] has affected both of us in the way that my mother has her own field which she used to cultivate with my father and harvest enough food for their family […] sometimes I don’t go to my field because I spend most of my time taking care of her. Which means this year there will be less harvest for both of us. Ultimately, it means we are going to have less food in our homes.’ (C010, family caregiver, female, 25 years)

‘Sometimes when my uncle becomes unwell, I do not go to work because his illness requires me to carry him on bicycle and escort him to the hospital, so I make sure that I help him.’ (C014, family caregiver, male, 21 years)

‘It affects us very much because he has children who are still going to school and usually when the bread winner, the one who provides school fees, food and clothing is unable to provide because he is sick, it affects the whole household. It really affects us.’ (C012, family caregiver, male, 26 years)

#### Theme 3: Consequences on caregiver well-being

**Sub-theme 3.1: Spiritual:** Caregivers who provided PPC without assistance reported that their caregiving responsibilities prevented them from attending religious services at the mosque or church. Some also explained that they were no longer able to participate in other social and community activities because of the demands of caregiving:

‘This part also has been affected for both of us, because both of us we don’t go to pray, for me I cannot go and leave him alone which means I don’t go to pray, and for him he can pray in his heart, but he cannot manage to go and pray together with other people.’ (C005, family caregiver, female, 53 years)

**Sub-theme 3.2: Psychosocial:** In addition to worries about the health of their older family member, caregivers also expressed concern for their own family and health, fearing that if they themselves became ill, the situation would spiral out of control as there would be no one to come to their aid:

‘I just ask God to protect me so I do not get ill, so that I can take care of my mother. Because if I get sick it means the whole family will suffer, including the children. Nobody will take care of them.’ (C10, family caregiver, female, 25 years)

‘There are more children whom am taking care of and I should take care of the mum since now she is also like a child. So taking care of the children, mum and the family, not easy.’ (C011, family caregiver, female, 29 years)

#### Theme 4: Expressed needs for fulfilling caregiver roles

**Sub-theme 4.1: Need for person-centred care:** Beyond the challenges encountered while providing PPC to older people, caregivers also described unmet needs related to their own health and well-being. For example, they emphasised the need for more time during clinical consultations to ask questions, discuss their concerns and receive guidance from healthcare professionals:

‘The doctors should ask patients or caregivers how we are staying with the patient. Like, “does he have concerns or worries”?’ (C13, family caregiver, female, 59 years)

**Sub-theme 4.2: Strengthened community-based primary care services:** They also emphasised the need for primary care services to be available closer to home, as most participants had to travel to the district hospital for treatment:

‘It would have been better if our health centre could have the medicine for high blood pressure so that we can easily collect the medicine for my mother. The health centre is closer than district hospital. We have problems to get transport.’ (C10, caregiver, female, 25 years)

**Sub-theme 4.3: Availability of essential medicines:** Finally, they stressed the need for essential medicines to be consistently available at the hospital, so they would not have to purchase them out-of-pocket:

‘They should be providing drugs consistently.’ (C008, caregiver, female, 60 years)

## Discussion

This study demonstrates that family caregivers of older people living with multimorbidity experience interconnected financial, social and emotional challenges that extend beyond the provision of day-to-day care. These findings provide important evidence for the development of PPC services in Malawi by highlighting the central role of family caregivers in supporting older people as they navigate fragmented health and social care systems.

Evidence demonstrates the positive impact of caring for older people with multimorbidity in high-income settings,^22,35,36^ where a range of support services, such as day care, pensions and disability grants, residential facilities, and mobility or disability equipment are available to meet PPC needs. In contrast, such services are often limited or absent in LMICs. Findings from this study revealed that primary palliative caregiving for an older person with multimorbidity in Malawi brings significant disruptions to family life, including financial strain and its consequences, such as household food insecurity, school dropout, and emotional and social distress. These findings align with previous studies showing that caregivers of patients with serious illnesses experience substantial burden.^17,23^ The study also extends the palliative care literature in SSA, which has predominantly focused on HIV and cancer, by examining caregiving in the context of ageing and multimorbidity.^37^ Such needs are likely to be amplified in the context of multimorbidity, where the absence of integrated or dedicated health care pathways necessitates multiple hospital visits particularly in settings like ours, where primary health centres often lack both the expertise and the essential medicines and supplies to manage multimorbidity effectively.^38^

Viewed through the illness-specific work domain of the Lines of Work framework,^39,40^ financial hardship emerged as a consequence of fragmented chronic care pathways rather than isolated household poverty. Long travel distances to facilities providing chronic disease services, together with frequent medicine stock-outs requiring out-of-pocket purchases, substantially increased the workload and economic burden borne by families.^41^ Similar financial pressures have been reported among caregivers of patients with advanced cancer,^10,19,42^ suggesting that inadequate access to affordable care is a common mechanism through which serious illness generates caregiver burden. These findings reinforce calls for decentralised chronic care, strengthened medicine supply systems and social protection measures to reduce the financial consequences of caregiving.^38^

Within the everyday work domain,^39^ caregiving disrupted households’ capacity to maintain livelihoods. Reduced participation in farming and income-generating activities contributed to food insecurity, illustrating how illness-related demands extend beyond health to affect household resilience.^43^ These findings support evidence from other LMICs that caregiving and poverty are mutually reinforcing,^44^ and suggest that palliative care policies should be complemented by social protection interventions that enable families to sustain livelihoods while providing care.^45^

Emotional work^39^ was strongly gendered, with women assuming most caregiving responsibilities, reflecting broader social norms documented across LMICs. The cumulative demands of illness-specific and everyday work appeared to intensify emotional strain, reinforcing the multidimensional nature of caregiver burden.^46^ These findings support previous recommendations that caregiver training and psychosocial support should be integrated into PPC,^47,48^ while policies should also promote greater male participation in caregiving.^49^

Similarly, studies have shown that the practice of PCC can reduce the burden on caregivers of people with serious illnesses.^50,51^ Evidence also highlights the value of integrated care for people with multimorbidity, as it better accommodates the needs of both patients and caregivers.^52,53,54^ The theory and evidence underpinning models of person-centred care in serious illness have largely come from Western high-income countries.^55^ This study adds to the emerging evidence from the global majority.^56^ Furthermore, the novel data in our study may inform new models of PPC, which currently lack a sound evidence base. Development of plausible models requires adherence to established consensus definitions of PPC in Africa and articulation of logic models to provide clarity in context and mechanisms.^30^

Further research is needed to design and evaluate effective interventions to care for older people with multimorbidity, with a focus on improving physical accessibility, strengthening support for HBC and advancing integrated, person-centred approaches. By addressing the complex needs of this population, such interventions have the potential to enhance quality of care while alleviating the challenge on both patients and their caregivers.

### Strengths and limitations

This study demonstrates several strengths, including the generation of diverse perspectives from family caregivers recruited at both a primary-level health centre and a primary-level clinic within a secondary-level district hospital. Furthermore, Mangochi’s peri-urban context meant that some participants brought experiences shaped by urban settings, while others reflected rural life, thereby enhancing the breadth of perspectives. Such diversity deepens the understanding of primary palliative caregiving by highlighting experiences from multiple angles. However, the study has limitations. It reports qualitative data from a relatively small group of participants in a single district of Malawi, which restricts transferability of findings to similar settings across Malawi and beyond.

## Conclusion

Family caregivers are indispensable providers of PPC for older people living with multimorbidity in Malawi, but they face considerable financial, physical, psychosocial and spiritual challenges that compromise both caregiver and patient well-being.

These findings suggest that effective PPC for older people living with multimorbidity requires interventions that extend beyond symptom management to address the practical and emotional realities of family caregiving and recognise caregivers as partners in care. Integrated, person-centred models of care, supported by stronger primary health care systems and social protection mechanisms, have the potential to improve outcomes for both patients and caregivers. Routine caregiver assessment should be incorporated into PPC consultations to identify caregiver burden and unmet needs, with timely referral for psychosocial support where appropriate. Health systems should strengthen decentralised community-based PPC services, improve the availability of essential medicines at primary health care facilities and expand integrated PCC services closer to people’s homes to reduce travel costs and the caregiving burden. Our work informs policymakers to consider social protection measures, caregiver education and community support programmes that strengthen family resilience and sustain caregiving. The data provide novel evidence from Malawi to inform policy, service delivery and future intervention development for older people living with multimorbidity and their family caregivers across SSA.
